# Activities of Serum Ferritin and Treatment Outcomes Among COVID-19 Patients Treated With Vitamin C and Dexamethasone: An Uncontrolled Single-Center Observational Study

**DOI:** 10.7759/cureus.11442

**Published:** 2020-11-11

**Authors:** Hemanth Reddy Burugu, Venkataramana Kandi, Lakshmi Venkata Simhachalam Kutikuppala, Tarun Kumar Suvvari

**Affiliations:** 1 Medicine, Prathima Institute of Medical Sciences, Karimnagar, IND; 2 Clinical Microbiology, Prathima Institute of Medical Sciences, Karimnagar, IND; 3 Medicine, Konaseema Institute of Medical Sciences and Research Foundation (KIMS & RF), Amalapuram, IND; 4 Public Health, Rangaraya Medical College, Kakinada, IND

**Keywords:** covid-19, dexamethasone, vitamin c, serum ferritin, ct-imaging, lungs, sars-cov-2

## Abstract

Background

The fatal outcomes by COVID-19 are accompanied by cytokine storm syndrome, and thereby it is reported that disease severity is dependent on the cytokine storm syndrome. This cytokine storm can be assessed by evaluating the serum ferritin levels. The objective of this study was to assess the activities of serum ferritin and treatment outcomes among COVID-19 patients who were treated with dexamethasone and vitamin C.

Materials and methods

A single-center, prospective, observational study was conducted among SARS-CoV-2 infected patients from July 2020 to August 2020. The diagnosis was confirmed by real-time polymerase chain reaction (RT-PCR) and computed tomography (CT) imaging of the lungs. Serum ferritin levels were compared with the treatment outcomes of COVID-19 positive patients who were treated with dexamethasone and vitamin C.

Results

A total of 50 COVID-19 patients were included in the study. The mean age was 41.70 years. The recovery rate (94%) was remarkably high and is a good sign of COVID 19 treatment with vitamin C and dexamethasone as key modalities. The mean serum ferritin levels among recovered and expired patients were 478.81 ng/ml and 1410 ng/ml, respectively.

Conclusion

The serum activities of ferritin were markedly increased in COVID-19 patients who could not survive as compared to the patients who finally recovered from the infection.

## Introduction

COVID-19 caused by SARS-CoV-2 was declared as a public health emergency on January 30, 2020. The outbreak was declared as a pandemic on March 11, 2020, by the World Health Organization (WHO). The primary mode of spread is through close contact and via respiratory droplets produced from coughs or sneezes. The SARS-CoV-2 main target is binding to the angiotensin-converting enzyme 2 (ACE2) receptors, which are most commonly present in the lung, kidney, and gastrointestinal tract [1].

SARS-CoV-2 infection, especially in older patients and those with pre-existing illness, can progress to severe disease with critical respiratory symptoms [2]. Therefore, early recognition of severe forms of COVID-19 is essential for the timely triaging of patients [2]. Ferritin is a crucial mediator of immune dysregulation, contributing to the cytokine storm under extreme hyperferritinemia via direct immune-suppressive and pro-inflammatory effects [3]. 

Elevated levels of ferritin, or hyperferritinemia, indicate the viral or bacterial load in the body. Hyperferritinemia, or hyperferritinemic syndrome, is a condition activating macrophages to secrete cytokines, causing a cytokine storm in severe cases, which can be a sign of severe disease [4]. The fatal outcomes by COVID-19 are accompanied by cytokine storm syndrome. It is thereby reported that disease severity depends on the cytokine storm syndrome. This cytokine storm can positively be assessed by evaluating the serum ferritin levels [5].

Ideally, serum ferritin levels might be a crucial factor influencing the severity of COVID-19. Many individuals with diabetes exhibit elevated serum ferritin levels, facing a higher probability of experiencing severe complications from COVID-19 [6]. The median values of serum ferritin levels from a few recent studies exceeded the upper limit of detection in the COVID-19 patients during all the days of hospitalization, suggesting that ferritin levels increased incessantly throughout the hospital stay [7].

In this study, we assessed the correlation between serum ferritin and severity and mortality of COVID-19 and treatment outcome with dexamethasone and vitamin C in COVID-19 positive patients. We aimed to evaluate the serum concentrations of ferritin and correlate it with the clinical outcomes.

## Materials and methods

A single-center, prospective, observational study was conducted among COVID-19 positive patients at a tertiary care hospital from July 2020 to August 2020 (2 months). All the patients included in the study were real-time polymerase chain reaction (RT-PCR) positive and had typical Computed tomography (CT) radiological features suggestive of COVID-19. The age range of the patients included in the study was between 25-50 years and written informed consent was obtained from all the study participants and confidentiality of information was assured to them. This study was approved by the Institutional Ethical Committee of the Prathima Institute of Medical Sciences, Telangana. The patients who had severe co-morbidities and were critically ill at the time of presentation and patients below 25 or above 50 years of age were excluded from the study.

The socio-demographic and clinical profile data were collected as shown in Table 1. A blood sample was collected from all the patients and sent for laboratory investigations; exclusively serum ferritin levels were compared with the treatment outcomes of all the patients who were treated with dexamethasone and vitamin C. The serum ferritin level was determined by chemiluminescence immunoassay (CLIA).

Statistical analysis

The data were expressed as mean or percentage. We compared the categorical variables between the two groups using Pearson's chi-square test. A p-value of < 0.05 was considered statistically significant. All statistical analyses were performed using the IBM Statistical Package for the Social Sciences (SPSS) version 24 (IBM Corp., Armonk, NY).

## Results

A total of 50 COVID-19 positive patients were included in the study. The mean age was 41.70 + 7.106. The serum ferritin levels were measured by the chemiluminescence assay (CLIA). Seventy-four percent (74%) of the participants are males, and 34% are females. Forty-six percent (46%) of the participants are between the ages of 45 and 50. Twenty-eight percent (28%) of the patients have co-morbidities, in which a combination of hypertension and diabetes mellitus is the most common. The serum ferritin levels of 29 patients (58%) are greater than 300. Forty-seven patients (94%) recovered, and three patients (6%) died during the study, which was regarded as the final outcome (Table 1).

**Table 1:** Demographic details, serum ferritin levels, and the final outcome of the patients (n=50)

		No. of. Individuals	Percentage %
Gender	Male	37	74%
	Female	13	26%
Age	25 – 35	12	24%
	36 – 44	15	30%
	45 – 50	23	46%
Co-morbidities	Only hypertension	3	6%
	Only diabetes mellitus	5	10%
	Hypertension and diabetes	4	8%
	Hyperthyroid	1	2%
	Hypothyroid	1	2%
Serum ferritin levels	Less than/equal to 300	21	42%
	Greater than 300	29	58%
Final outcome	Died	3	6%
	Recovered	47	94%

The recovery rate (94%) was very high and is a good sign of COVID-19. Low levels of serum ferritin were observed among them, where treatment with vitamin C and dexamethasone as key modalities. The mean serum ferritin levels among patients who survived and those who expired were 478.81 ng/ml and 1410 ng/ml, respectively (Table 2).

**Table 2:** Mean and p-values of serum ferritin among recovered and death patients (n=50)

	Recovered (n=47) (Mean + SD)	Death (n=3) (Mean + SD)	P-value
Serum ferritin levels (ng/ml)	478.81 + 424.69	1410 + 370.7	0.806

## Discussion

Ferritin (storage protein) is involved in iron metabolism, which contains L and H subunits expressed in the lung and heart, respectively. The H subunit involves the inflammatory mechanism by participating in myeloid and lymphoid cell proliferation and stimulating TIM-2, a specific ferritin receptor. H-ferritin plays a major role in immunomodulatory and pro-inflammatory activities by activating several inflammatory mediators such as IL-1β. Ferritin was found only in the lymph node B area, indicating its role as an antigen, which stimulates macrophage activation related to hyperferritinemia [8].

Previous research studies have revealed the role of hyperferritinemia in assessing the severity of disease among COVID-19 patients. The possible mechanisms include increased ferritin synthesis by proinflammatory cytokines such as tumor necrosis factor-alpha (TNF-α), interleukin 6 (IL-6), and IL-lβ. This leads to increased inflammation that causes cell damage and the release of ferritin [9]. Hyperferritinemia patients were found to have increased mortality risk; it is not clear whether it is a coincidence or due to viral pathogenesis [10].

Through this observational study, we focused on the activities of serum ferritin as a vital SARS-CoV-2 infection marker among COVID-19 patients. The serum activities of ferritin were markedly increased in the patients who could not survive the treatment as compared with patients who finally recovered from the infection. This could be attributed to more severe secondary bacterial infection and increased inflammation leading to exacerbated COVID-19. The reasons for increased ferritin can be due to viral or bacterial infection, hemochromatosis, and long-term transfusion [11]. When secondary microbial infections occur alongside a pre-existing viral infection, an increase in the serum activities of ferritin was commonly observed, which was attributed to the release of iron from the reticuloendothelial system [12].

Patients with bacterial infection are found to have higher ferritin levels as compared to patients with a viral infection, which is evident through some previous studies [13]. In addition, the elevation of serum ferritin levels is predictive of a poor outcome in hospitalized patients with influenza infection [14]. In the present study, almost all COVID-19 patients showed increased serum ferritin levels, but the activities of serum ferritin extremely increased in the severely ill COVID-19 group, who could not survive. The increased ferritin might indicate a severe secondary bacterial infection in COVID-19 and might be utilized as a marker of poor prognosis [15].

From a randomized controlled trial in critically ill patients with sepsis-induced acute respiratory distress syndrome (ARDS) (n = 167), it is observed that the administration of intravenous (IV) vitamin C 200 mg/kg per day for four days lowered 28-day mortality in the treatment group (29.8% vs. 46.3%; P = 0.03), coinciding with more days alive and free of the hospital and intensive care unit (ICU) admission [16]. From a study conducted in patients hospitalized with COVID-19, the use of dexamethasone resulted in a 28-day decreased mortality among those who were receiving either invasive mechanical ventilation or oxygen alone at randomization [17]. The recovery rate in this study seems to be high due to treatment with a high dose of vitamin C and dexamethasone and because none of the patients recruited in the study were >65 years of age, a well-established predisposing factor for severe and life-threatening COVID-19.

Limitations of the study

The major concern this study had was the absence of a comparator or a control group. The outcome of the patients could not be attributed either to the activities of serum ferritin or the treatment regimen that included dexamethasone and vitamin C, which was given universally for all the COVID-19 patients.

## Conclusions

Increased activities of serum ferritin were observed among most COVID-19 patients. The serum activities of ferritin were extremely elevated among the COVID-19 patients who could not survive the treatment as compared to the recovered patients. The serum concentrations of ferritin could be used as a prognostic marker in the management of COVID-19 patients.
